# DeepDrug3D: Classification of ligand-binding pockets in proteins with a convolutional neural network

**DOI:** 10.1371/journal.pcbi.1006718

**Published:** 2019-02-04

**Authors:** Limeng Pu, Rajiv Gandhi Govindaraj, Jeffrey Mitchell Lemoine, Hsiao-Chun Wu, Michal Brylinski

**Affiliations:** 1 Division of Electrical & Computer Engineering, Louisiana State University, Baton Rouge, LA, United States of America; 2 Department of Biological Sciences, Louisiana State University, Baton Rouge, LA, United States of America; 3 Division of Computer Science and Engineering, Louisiana State University, Baton Rouge, LA, United States of America; 4 Center for Computation & Technology, Louisiana State University, Baton Rouge, LA, United States of America; Hebrew University of Jerusalem, ISRAEL

## Abstract

Comprehensive characterization of ligand-binding sites is invaluable to infer molecular functions of hypothetical proteins, trace evolutionary relationships between proteins, engineer enzymes to achieve a desired substrate specificity, and develop drugs with improved selectivity profiles. These research efforts pose significant challenges owing to the fact that similar pockets are commonly observed across different folds, leading to the high degree of promiscuity of ligand-protein interactions at the system-level. On that account, novel algorithms to accurately classify binding sites are needed. Deep learning is attracting a significant attention due to its successful applications in a wide range of disciplines. In this communication, we present DeepDrug3D, a new approach to characterize and classify binding pockets in proteins with deep learning. It employs a state-of-the-art convolutional neural network in which biomolecular structures are represented as voxels assigned interaction energy-based attributes. The current implementation of DeepDrug3D, trained to detect and classify nucleotide- and heme-binding sites, not only achieves a high accuracy of 95%, but also has the ability to generalize to unseen data as demonstrated for steroid-binding proteins and peptidase enzymes. Interestingly, the analysis of strongly discriminative regions of binding pockets reveals that this high classification accuracy arises from learning the patterns of specific molecular interactions, such as hydrogen bonds, aromatic and hydrophobic contacts. DeepDrug3D is available as an open-source program at https://github.com/pulimeng/DeepDrug3D with the accompanying TOUGH-C1 benchmarking dataset accessible from https://osf.io/enz69/.

## Author summary

Small organic ligands bind to the locations of chemical specificity and affinity on their protein targets, called binding sites. A typical ligand-binding site is a small pocket formed by a few residues while the remaining protein structure acts as a framework providing the correct orientation of binding residues. Annotating ligand-binding sites is complicated by a fact that the same small molecule often binds to similar pockets but located in different proteins. In order to improve the detection and classification of binding pockets in proteins, we developed a new computational tool, DeepDrug3D. Our algorithm employs a convolutional neural network, a class of deep learning already commonly used in visual imagery analysis, recommender systems, and natural language processing. DeepDrug3D is able to accurately classify binding sites by learning the patterns of specific molecular interactions between ligands and their protein targets, such as hydrogen bonds, aromatic and hydrophobic contacts. Although the current proof-of-concept implementation is limited to a few most abundant functional classes, the repertoire of pocket types handled by DeepDrug3D will significantly be expanded in the near future.

This is a *PLOS Computational Biology* Software paper.

## Introduction

Proteins constitute a diverse group of biological macromolecules essential for the vast majority of processes in living organisms. Particularly, interactions between proteins and small organic ligands are indispensable to many cellular functions on account of their significant roles in a wide variety of biological pathways. Experimental techniques such as X-ray crystallography, nuclear magnetic resonance spectroscopy, and cryo-electron microscopy are used to uncover intricate mechanisms of ligand-protein interactions at the atomic level. The resulting wealth of structural data collected for a large number of organisms across all domains of life are available from the Protein Data Bank (PDB) [1]. Parallel to experimental methods, computational approaches to detect and analyze ligand-protein interactions notably contribute to numerous resources cataloging biological complexes, such as sc-PDB [2], BioLiP [3], PDBbind [4], Relibase [5], and the Protein-Ligand Interaction Clusters, or PLIC, database [6]. Despite a continuous growth of the structural information in the PDB [7, 8], ligand binding to many hypothetical proteins can only be inferred computationally [9]. These predictions are typically obtained by using either global structures or confined regions on the protein surface, where putative ligands bind, referred to as binding sites or binding pockets [10]. In recent years, approaches employing comparative binding site analysis have gained traction in structural bioinformatics because these techniques are capable of revealing ligand-binding similarities independently of evolutionary relationships between proteins [11, 12]. Since unrelated proteins may bind the same type of ligand molecules [13], binding site classification is an important tool to support modern drug design focused on polypharmacology, drug repurposing, and the prediction of drug side-effects [14, 15].

Nucleotide-binding proteins are notable examples of dissimilar proteins interacting with similar ligands [16]. Abundant in biological cells, nucleotides play central roles in metabolism, synthesis, active transport, cell signaling, and the maintenance of cell structure. Because of their critical functions, nucleotide-binding sites are one of the largest class of drug targets [17]. On that account, the accurate detection, classification, and characterization of nucleotide-binding proteins and pockets are of paramount importance not only for the systems-level protein function annotation [18, 19], but also for the rational design of competitive inhibitors for pharmacotherapy [20, 21]. Another diverse class of proteins binding the same ligand are hemeproteins containing a heme prosthetic group. These macromolecules are ubiquitous in biological systems contributing to various biological activities, including oxygen transport, electron transfer, ion channel chemo-sensing, circadian clock control, microRNA processing, and transcriptional regulation [22]. Although many hemeproteins are already well-characterized, some orphan proteins of unknown function may bind heme. For instance, heme was identified as a physiological ligand of orphan nuclear receptors REV-ERBα and REV-ERBβ, extending the collection of known ligands of the human nuclear receptor family beyond endocrine hormones and dietary lipids [23]. Despite the fact that the PDB contains about 3,600 proteins complexed with some form of heme, new binding modes of heme are still being discovered [24]. Therefore, a reliable classification and characterization of binding sites in hypothetical proteins may support studies focused on the identification of physiological ligands and their binding modes.

Many structure-based approaches to classify ligand-binding sites build on pocket similarity detection [25, 26]. Traditional methods commonly employ algorithms to solve the clique and assignment problems, conduct hashing and sorting, and perform the rotational and translational search [15]. For instance, G-LoSA detects similar pockets and constructs local structure alignments by combining the assignment algorithm with the clique detection [27]. G-LoSA was recently shown to outperform other algorithms to match binding sites [28]. Other examples are SOIPPA (Sequence Order Independent Profile-Profile Alignment) [29] and its successor, SMAP [30], representing amino acids with the Delaunay tessellation of Cα atoms. Benchmarking of SMAP and SOIPPA against a large dataset of adenine-binding sites showed that these algorithms effectively identify known sequence and structural homologs within the same superfamily according to the Structural Classification of Proteins, or SCOP [31]. IsoMIF Finder is an online server to identify similarities between binding sites represented as molecular interaction fields (MIFs) [32]. This approach utilizes six chemical probes to identify geometrically and chemically equivalent sections of protein cavity pairs by detecting their subgraph isomorphisms [33]. Finally, machine learning-based techniques, such as *e*MatchSite, offer not only a high accuracy of binding sites comparison against diverse datasets, but also a remarkable tolerance to structure distortions in computer-generated protein models [11, 34]. The detection of similar binding sites is often used in projects focused on polypharmacology [35, 36] and drug repositioning [37, 38]. Nonetheless, many of these tools are template-based decreasing the coverage of suitable targets, employ handcrafted feature vectors, and may not perform well on unseen data.

In principle, these issues can be addressed by using advanced deep learning methods to compare and classify ligand-binding sites in proteins. Deep learning provides the-state-of-the art performance in computer vision [39], natural language processing [40], and other research areas [41, 42]. The convolutional neural network (CNN) and its variations are by far the most popular deep learning algorithms. For instance, ImageNet [43] and Deep Residue Learning [44] show an unparalleled performance in image classification. Not surprisingly, deep learning approaches hold significant promise for applications in biology and biomedicine. DeepSF is a recently developed protein fold predictor employing a 1D-convolution neural network [45]. It outperforms profile-profile alignment methods such as PSI-BLAST [46] and HHsearch [47]. A CNN-based protein-binding site predictor, DeepSite, represents protein structures as 3D images comprising voxels assigned a series of pharmacophoric properties [48]. DeepSite was shown to be more accurate than Fpocket [49] and Concavity [50] against the sc-PDB database of binding sites [2]. Another example is LigVoxel deploying CNNs to predict ligand chemical properties, such as the occupancy, aromaticity and donor-acceptor positions, for a given protein pocket [51]. Importantly, benchmarking calculations against the Astex diverse set [52] demonstrate that fields predicted by LigVoxel largely overlap with compounds bound to the target pocket, which allows to recover the majority of the original ligand crystal poses. Finally, K_DEEP_ is a protein-ligand affinity predictor built on CNNs [53], representing molecular structures as voxels containing several pharmacophore-like attributes. K_DEEP_ predicts binding affinities with the Pearson correlation coefficient of 0.82 and the root-mean-square error of 1.27 in p*K* units against experimental values provided by PDBbind [4], thus it is significantly more accurate than other methods.

In this communication, we describe DeepDrug3D, a new method to classify ligand-binding sites in proteins with CNNs. As a proof of concept, we present the results of classification benchmarks conducted for two the most abundant in the PDB classes of pockets, nucleotide- and heme-binding, against a diverse control dataset of pockets. Further, independent sets of steroid-binding pockets and peptidase enzymes are used to evaluate the performance of DeepDrug3D on unseen data. This approach is versatile and will be expanded in the near future to include a variety of classes of druggable pockets.

## Design and implementation

DeepDrug3D employs a multi-layer CNN to classify binding pockets. In this section, we first present a procedure to generate 3D pocket grids and their voxel representations, followed by the description of the CNN structure and the class-activation map. Next, we provide details on datasets created to train and validate DeepDrug3D, assessment criteria, and other approaches used in comparative benchmarks.

### Generation of 3D pocket grids and voxels

**Fig 1:**
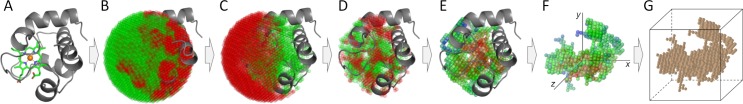
Voxelization of ligand-binding pockets. (**A**) Starting from a ligand-protein complex, a sphere centered on the geometric center of the ligand is created and filled with a 3D grid. Grid points (**B**) overlapping with the protein, (**C**) too far away from the protein, and (**D**) disconnected from the main grid structure are removed. Points to be removed are shown in red in **B**-**D**. Subsequently, (**E**) statistical potentials for ligand-protein interactions are calculated at each grid point and (**F**) the pocket principal axes are aligned to the Cartesian axes. (**G**) The voxel representation of a ligand-binding pocket is used as an input in deep learning.

Deep learning requires ligand-protein complexes to be converted to uniform structures. Although volume elements, or voxels, are naturally the best candidate to represent molecular structures, a simple atom-based voxelization of complexes is not appropriate because of different sizes of proteins and bound ligands. Therefore, ligand-protein complexes in DeepDrug3D are represented by fixed-size 3D pocket grids, which are subject to voxelization in order to generate input data for the CNN. The flowchart for the generation of 3D pocket grids followed by their voxelization is presented in Fig 1. Given a ligand-protein complex (Fig 1A), a spherical grid with a 15 Å radius and a 1 Å spacing centered on the geometric center of the ligand is generated. A three-step refinement procedure is then applied in order to obtain a precise representation of the actual pocket by removing unnecessary grid points. First, excluded volume points, defined as those located within 2 Å from any protein atom, are eliminated (red points in Fig 1B). Second, points outside of the convex hull of the protein structure, which is considered as the union of envelopes of all protein atoms, are removed (red points in Fig 1C). Third, discarding points detached from the largest connected component in the pocket grid (red points in Fig 1D) creates the final, continuous grid structure.

In addition to the spatial information conveyed by grid point coordinates, the physicochemical properties of binding pockets are characterized with a distance-scale finite ideal-gas reference (DFIRE) potentials [54]. For each grid point in the refined pocket grid, we calculate interaction energy values against all protein atoms for the following 14 atom types according to SYBYL [55]: carbon (*C*.2, *C*.3 and *C*.ar), nitrogen (*N*.2, *N*.4, *N*.am, *N*.ar and *N*.pl3), oxygen (*O*.2, *O*.3 and *O*.co2), phosphorous (*P*.3), sulfur (*S*.3), and halogens (*F*). An example of a potential calculated for *C*.ar over all grid points is shown in Fig 1E, where the red color indicates a low (preferable) interaction energy, the blue color denotes a high (poor) interaction energy, and values in between are green. In order to standardize the input orientation for the CNN, 3D pocket grids are uniquely positioned in space so that their centers are located at x=y=z=0 and the longest, middle and shortest principal axes calculated for binding residues align to x, y and z Cartesian axes, respectively (Fig 1F). This unique orientation is important because pocket classification is treated as a computer vision problem.

In the last step, the voxelization of 3D pocket grids is performed (Fig 1G). The space is discretized into 32×32×32 voxels, where each voxel is a pocket grid point assigned with DFIRE potentials. This voxel size was selected to engird the majority of ligands and their binding residues used in this study. The voxel representation can be considered as a 3D image with 14 channels corresponding to the interaction energies of various ligand atom types with protein residues instead of the conventional RGB color channels.

### Convolutional neural networks

CNNs are similar to traditional artificial neural networks (ANNs). Each neuron takes some input x, calculates the dot product of x and a set of weights $w_{i}$, adds the bias b, and applies a non-linear activation function f. Thus, the output of a neuron φ can be expressed as:

$$\varphi =f\left(\sum_{i}w_{i}x_{i}+b\right)$$ Eq 1

where the results of each input $x_{i}$ is summed up as the final output for the entire layer of neurons.

Complicated and noisy input is poorly handled by ANNs because these networks cannot take advantage of the spatial invariance. For example, given the image of a dog, where the dog can appear anywhere in the image, ANNs learn independent weights at each spatial location. As a result, a group of neurons receiving inputs from the lower left corner of the image need to learn the representation of a dog independently from the group of neurons in the upper left corner. Such networks require enough images of dogs at all possible locations to be effectively trained. CNNs solve this problem by repetitively applying blocks of neurons across the entire image space, allowing to share the weights. Consequently, CNNs offer the desirable spatial invariance of the input data.

Inspired by a classical 3D object classification network, VoxNet [56], a CNN implemented in DeepDrug3D employs several types of layers. *Convolutional layers* calculate the dot product of the input and weights updated as a part of the training process. These layers are capable of extracting local features since the convolutional kernel size is usually much smaller than the input data size. Although the preceptive fields of filters are restricted to local features only, these attributes are shared by all kernels to capture both local and global features. By hierarchically arranging different convolution layers, CNNs learn different levels of features such as edges, blobs, colors, species, etc. The hyperparameters of convolutional layers include number of feature maps, filter kernel size, stride and padding. *Activation layers* add the non-linearity to the model by applying an activation function f to the output of the previous layer. The most common activations are sigmoid, hyperbolic tangent, softmax, ReLu (a rectified linear unit), and leaky ReLu, which overcomes the so-called dying ReLu problem [57]. *Pooling layers* reduce the resolution of features by down-sampling the spatial dimension of feature maps. This procedure not only makes features more robust against the noise and distortions, but also helps reduce the number of parameters in the network. Two most commonly used pooling layers are max pooling and average pooling. *Fully connected* layers often serve as final layers in CNNs. They sum up weights of the previous layers determining a precise mix of features to achieve a specific target output. Output features of fully connected layers are based on all elements of all features of the previous layer. Finally, *other layers* include many functional layers, such as the dropout layer that helps overcome the overfitting problem by randomly selecting a portion of neurons to be activated.

**Fig 2:**
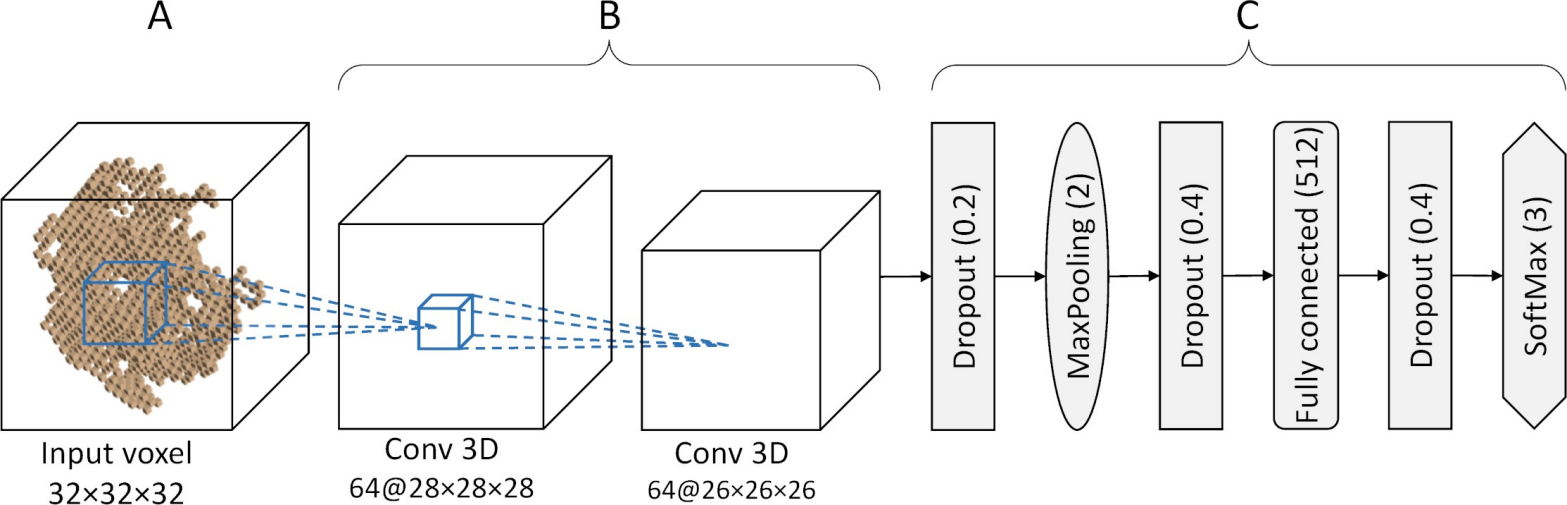
Structure of a convolutional neural network in DeepDrug3D. The network consists of (**A**) an input voxel followed by (**B**) two convolutional layers with leaky ReLu activation functions, and (**C**) a series of dropout, pooling, fully connected and softmax layers.

The architecture of a CNN implemented in DeepDrug3D is shown in Fig 2. The input voxel (Fig 2A) is followed by two consecutive convolutional layers with leaky ReLu activation functions (Fig 2B). The output from the second convolutional layer, 64 feature maps of size 26×26×26Å, are passed through a dropout layer, a maxpooling layer, and another dropout layer before entering the fully connected layer (Fig 2C). Since the network output are different ligand types, softmax is the final activation layer. Note that this architecture is similar to the VGG-network comprising multiple blocks of stacked convolutional layers followed by dropout and maxpooling layers [58]. However, since our voxel size of 32×32×32×14 is significantly larger than the typical image data used in computer vision, such a deep network architecture would be computationally unfeasible. Further, because of a relatively small number of samples, i.e. non-redundant binding sites in the PDB, a simpler architecture helps avoid overfitting, yet it still captures distinctive features to accurately classify binding pockets.

Hyperparameters important for training are the optimization algorithm, the batch size, the number of epochs, the learning rate for optimization, and the learning rate decay. DeepDrug3D employs the Adam optimization algorithm, which was shown to outperform other methods in terms of the robustness and stability [59]. The learning rate, the learning rate decay, and $\beta_{1}$ and $\beta_{2}$ hyperparameters of the Adam are set to 0.00001, 0, 0.9, and 0.999, respectively. We found empirically that different batch sizes of 16, 32, 64, and 128 yield a comparable performance, and 50 epochs are sufficient to reach the convergence. A 5-fold cross-validation of DeepDrug3D with a voxel size of 32×32×32×14 requires 3 hours on a Nvidia Tesla K20 GPU.

### Class activation maps

**Fig 3:**
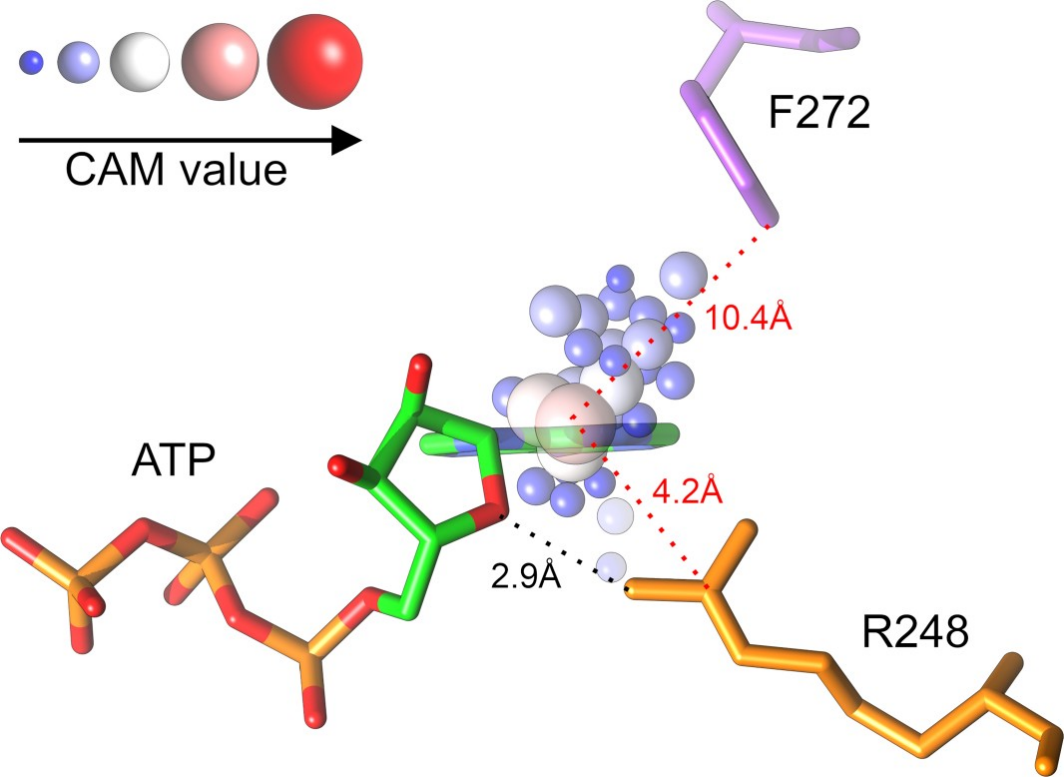
An example of class-activation map (CAM) grids within ligand-binding sites. ATP bound to the human TRPV4 ankyrin repeat domain is shown as sticks colored by atom type (C–green, O–red, N–blue, and P–orange). Selected grid points are represented by spheres whose size and color depend on the assigned CAM value according to the scale in the top left corner. Two residues are shown, R248 (orange) forming a hydrogen bond with the ribose moiety of ATP and a more distant residue F272 (purple). A dotted black line represents a hydrogen bond, whereas dotted red lines mark the distance between protein residues and the highest-scoring grid point.

Since not all binding regions contribute equally to the accurate pocket classification, the class-activation map, or CAM, analysis is used to identify the most discriminative sections of binding pockets. This technique was originally applied in image classification to detect discriminative image regions in a single forward pass, even including tasks the network was not originally trained for [60]. Briefly, the CAM explores the CNN localization ability by utilizing the global average pooling layer to identify regions in the original data that are important to distinguish between different classes. A 3D voxel version of the CAM is implemented in DeepDrug3D. After calculating the voxel-based CAM, only grid points whose CAM values are within the top 1% are retained, yielding around 300 points for a typical ligand-binding site. Next, each grid point is mapped to its closest protein residue within a distance of 5 Å. For a given binding residue, CAM values assigned from the surrounding grid points are added in order to compute a single CAM-score signifying the importance of that residue to the final classification. The CAM procedure is illustrated in Fig 3 for an ATP-binding site in the ankyrin repeat domain of the transient receptor potential cation channel subfamily V member 4 (TRPV4) [61]. A cluster of high scoring grid points are located around the nucleoside moiety of ATP with the highest scoring grid point assigned to a nearby amino acid R248. Using residue-based CAM-scores allows for the evaluation of individual binding residues with respect to their importance to the classification model. For instance, CAM-scores for hydrogen bonded residues, such as R248 in Fig 3, can be compared to CAM-scores assigned to residues not forming hydrogen bonds with bound ligands, such as F272 in Fig 3.

### Dataset of nucleotide- and heme-binding pockets

For training and cross-validation, the daTaset tO evalUate alGoritHms for binding site Classification, or TOUGH-C1, is compiled. TOUGH-C1 comprises nucleotide- and heme-binding pockets because a large number of complexes containing these ligands in the PDB [62] provide structurally diverse data that are sufficient to conduct statistically meaningful benchmarks. First, 261,063 ligand-protein structures have been extracted from the PDB as of November 2017. Subsequently, the kcombu program [63] was deployed to identify those complexes containing nucleotides and heme molecules. Specifically, we selected 15,203 proteins binding small molecules whose Tanimoto coefficient (TC) to ATP is ≥0.7 and 14,665 proteins binding small molecules whose TC to heme is ≥0.7. Clustering each group of complexes with the CD-HIT program [64] at a protein sequence identity threshold of 80% produced 1,553 nucleotide- and 596 heme-binding clusters. A representative complex from each cluster was selected to compile the dataset of nucleotide- and heme-binding pockets. The analysis of ligand-protein contacts in TOUGH-C1 complexes was conducted with the Ligand-Protein Contacts (LPC) software [65] in order to identify protein residues forming hydrogen bonds, aromatic and hydrophobic contacts with bound ligands.

### Dataset of steroid-binding pockets

In addition to the datasets of nucleotide- and heme-binding pockets, we prepared a smaller set of steroid-binding pockets similar to that reported previously [34]. These pockets are used as a negative set to further assess the false positive rate against an independent dataset. First, 518 proteins binding small molecules whose TC to 17β-estradiol is ≥0.7 were selected from the PDB. Clustering these complexes with CD-HIT at a protein sequence identity threshold of 80% produced 69 steroid-binding clusters. Representative complexes from each cluster are included in the TOUGH-C1 dataset as a non-redundant set of steroid-binding pockets.

### Control dataset

A control dataset of ligand-binding pockets was prepared from the TOUGH-M1 dataset, previously used to benchmark the performance of several pocket matching algorithms [28]. Those TOUGH-M1 complexes composed of proteins whose sequence identity to any nucleotide-, heme-, and steroid-binding protein is ≤40% and the Template Modeling (TM)-score [66] is ≤0.5 were selected. The TM-score is used to evaluate the global structure similarity between a pair of proteins; it ranges from 0 to 1 with a value of 0.5 indicating a statistically significant structure similarity [67]. We also excluded proteins binding ligands whose TC to ATP, heme, or 17β-estradiol is >0.5. This procedure resulted in a non-redundant and diverse set of 1,946 pockets included in the TOUGH-C1 dataset as the control dataset.

### Peptidase dataset

Finally, we prepared an independent dataset of peptidases, a diverse family of enzymes catalyzing the hydrolysis of peptide bonds [68]. First, ligand-bound structures of the five largest groups of peptidases, serine endopeptidases (the Enzyme Commission number, EC, 3.4.21, 4,057 entries), cysteine endopeptidases (EC 3.4.22, 1,233 entries), aspartic endopeptidases (EC 3.4.23, 1,764 entries), metalloendopeptidases (EC 3.4.24, 1,042 entries), and threonine endopeptidases (EC 3.4.25, 4,370 entries), were identified in the PDB. Similar to the other datasets, we clustered these complexes with CD-HIT at a protein sequence identity threshold of 80%. The resulting peptidase dataset comprises 124 serine endopeptidases, 83 cysteine endopeptidases, 44 aspartic endopeptidases, 75 metalloendopeptidases, and 28 threonine endopeptidases, totaling 354 complexes.

### Evaluation metrics

The performance of DeepDrug3D for nucleotide- and heme-binding pockets against the control dataset is evaluated with a 5-fold cross-validation, a common strategy used in machine learning to assess model generalization [69]. In the k-fold cross-validation, the entire dataset is first divided into k non-overlapping subsets, where the first subset is used as a validation set for a model trained on the remaining k-1 subsets. This procedure is repeated k times employing different subsets as the validation set. Averaging the performance obtained for all k subsets yields the overall performance with the estimated validation error of the model. Since our data is relatively balanced with respect to different classes, data augmentation is not required. For the peptidase dataset, the model is cross-validated by excluding one entire EC group during model training and then using the omitted pockets as the validation set. This procedure is repeated 5 times each time excluding a different group of peptidase enzymes.

Pocket classification is assessed by the accuracy (ACC), the precision or positive predictive value (PPV), the specificity or true negative rate (TNR), the sensitivity or true positive rate (TPR), and the fall-out or false positive rate (FPR):

$$ACC=\frac{TP+TN}{TP+FP+TN+FN}$$ Eq 2

$$PPV=\frac{TP}{TP+FP}$$ Eq 3

$$TNR=\frac{TN}{TN+FP}$$ Eq 4

$$TPR=\frac{TP}{TP+FN}$$ Eq 5

$$FPR=\frac{FP}{FP+TN}$$ Eq 6

where TP is the number of true positives (correctly recognized either nucleotide- or heme-binding proteins), and TN is the number of true negatives (correctly recognized control proteins). FP is the number of false positives (control proteins classified as either nucleotide- or heme-binding), and FN is the number of false negatives (either nucleotide- or heme-binding proteins classified as control). In addition, the overall classifier quality is assessed with the Receiver Operating Characteristic (ROC) analyzing a trade-off between the TPR and the FPR for varying decision threshold values [70]. The area under the ROC curve (AUC) helps select the best model considering the cost and the class distribution.

### Other methods to classify binding pockets

The performance of DeepDrug3D is compared to that of three different approaches to classify binding pockets based on local sequence and structure characteristics, and ligand docking. The local sequence-based method employs PROSITE, a database of protein domains, families and functional sites, and their associated patterns and profiles [71]. First, two groups of sequence signatures were selected from PROSITE, one for nucleotide-binding sites comprising 61 patterns identified with “NUCLEOTIDE”, “ATP”, “AMP”, “ADP”, “ADENINE” and “KINASE” keywords, and another for heme-binding sites comprising 23 patterns identified with “HEM/HEME”, “PORPHYRIN” and “GLOBIN” keywords. Next, the ScanProsite program [72] was used to classify nucleotide- and heme-binding proteins against the control dataset based on the presence of each signature. From the initial set of patterns, we selected those nucleotide- and heme-binding signatures yielding the Matthews correlation coefficient (MCC) [73] of ≥0.1. The MCC ranges from -1 (anti-correlation) to 1 (a perfect classifier) with values around 0 corresponding to a random guess:

$$MCC=\frac{TN\times TP-FP\times FN}{\sqrt{\left(TP+FP\right)\left(TP+FN\right)\left(TN+FP\right)\left(TN+FN\right)}}$$ Eq 7

where TP, TN, FP, and FN are defined in the previous section. Four discriminative nucleotide-binding signatures are: ATP/GTP-binding site motif A (P-loop) (PS00017, MCC = 0.404), protein kinase ATP-binding region (PS00107, MCC = 0.196), serine/threonine protein kinase active-site (PS00108, MCC = 0.178), and tyrosine protein kinase specific active-site (PS00109, MCC = 0.104). Six discriminative heme-binding signatures are: globin family (PS01033, MCC = 0.359), cytochrome P450 cysteine heme-iron ligand (PS00086, MCC = 0.264), catalase proximal heme-ligand (PS00437, MCC = 0.135), peroxidase proximal heme-ligand (PS00435, MCC = 0.121), protozoan/cyanobacterial globin (PS01213, MCC = 0.114), and cytochrome b5 family heme-binding domain (PS00191, MCC = 0.108). The final local sequence-based classifier scans target sequences for the presence of these discriminative PROSITE patterns; a protein is classified as nucleotide- or heme-binding if at least one respective signature is identified.

The local structure-based method to classify binding pockets employs G-LoSA, an algorithm to detect similar pockets from local structure alignments [27]. For each target in nucleotide-binding and control datasets, pocket similarities against nucleotide-binding sites are calculated excluding self-pairs. G-LoSA evaluates pocket similarity with a size-independent GA-Score ranging from 0 to 1. The ROC analysis is then performed using the average GA-Score as a decision threshold value. The same protocol is applied to classify heme-binding pockets against the control dataset. Note that since global sequence and structure similarities are eliminated in nucleotide-, heme-binding, and control datasets, the classification with G-LoSA is based entirely on pocket matching among proteins having different structures.

The last method uses molecular docking with AutoDock Vina [74] to conduct inverse virtual screening. 3D structures of ATP and heme obtained from DrugBank [75] were converted to the PDBQT format with Open Babel [76]. The structures of nucleotide-, heme-binding and control proteins were converted to the PDBQT format with MGL tools [77]. The optimal docking boxes, 19.6 Å for ATP and 21.3 Å for heme, were calculated from the radii of gyration of both molecules with the *e*BoxSize program [78]. Following the inverse virtual screening protocol, the ATP molecule is docked to nucleotide-binding and control proteins and the ROC analysis is then performed using the binding affinity reported by Vina as a decision threshold value. The same protocol is applied to evaluate the performance of molecular docking for heme-binding pockets based on the predicted binding affinity of the heme molecule to heme-binding and control proteins.

## Results

### Classification performance for nucleotide- and heme-binding pockets

**Fig 4:**
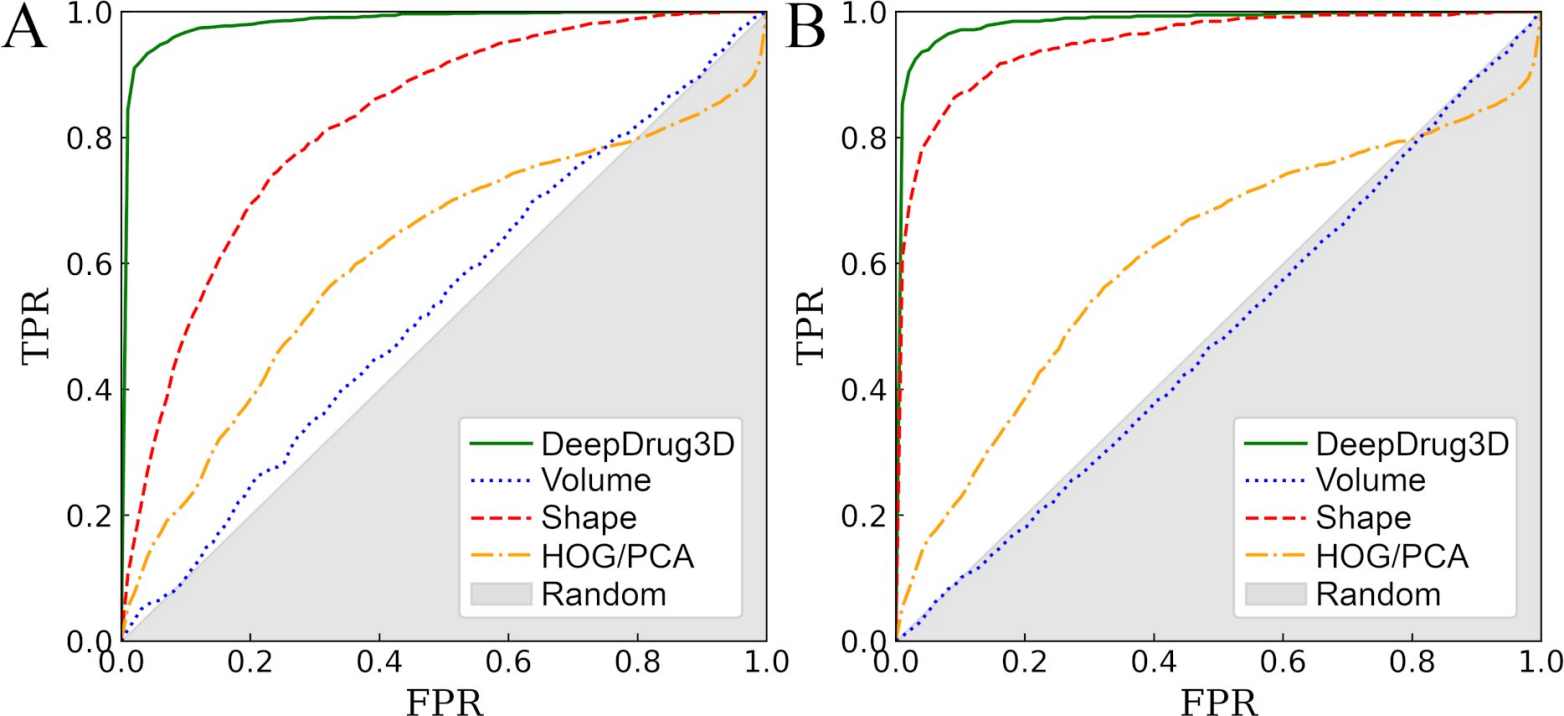
ROC plots evaluating the performance of various algorithms to classify ligand-binding sites. DeepDrug3D is compared to volume- and shape-based approaches, as well as a classifier employing the histogram of gradients with principal component analysis (HOG/PCA) for (**A**) nucleotide- and (**B**) heme-binding pockets. The *x*-axis shows the false positive rate (FPR) and the *y*-axis shows the true positive rate (TPR). The gray area represents a random prediction.

The overall performance of DeepDrug3D against the TOUGH-C1 dataset is assessed in Fig 4 and Tables 1 and 2. Encouragingly, DeepDrug3D classifies nucleotide-binding pockets with an AUC of 0.986 (Fig 4A) and heme-binding pockets with an AUC of 0.987 (Fig 4B). In addition to AUC values, Table 1 reports several other metrics derived from the confusion matrix. To fully appreciate these results, we compare DeepDrug3D with several other approaches. First, pockets are classified based on their volumes with a simple linear discriminant analysis (LDA) model trained on the volumes of 3D pocket grids. This approach yields a random performance because the volumes of nucleotide- (3,586 Å^3^ ±1,282) and heme-binding sites (4,496 Å^3^ ±1,263) are quite comparable to the volumes of control pockets (3,634 Å^3^ ±1,387). Next, we evaluate the accuracy of a shape-based approach by changing the voxel size from 32×32×32×14 to 32×32×32×2, which is equivalent to replacing interaction potentials with a binary occupancy (2 channels instead of 14). This representation of binding pockets contains only the shape information without any physicochemical properties. Interestingly, the AUC for this shape-based classifier is 0.824 for nucleotide- and 0.952 for heme-binding pockets. These results can be attributed to the fact that binding nucleotides and heme require pockets having different shapes than those binding control molecules. Indeed, the average radius of gyration for nucleotide, heme, and control ligands in the TOUGH-C1 are 12.5 Å ±1.0, 14.3 Å ±0.7, and 11.3 Å ±5.4, respectively, therefore control ligands are slightly smaller, yet more heterogeneous in shape.

**Table 1:** Performance of various algorithms to classify nucleotide-binding sites. DeepDrug3D is compared to volume- and shape-based approaches, a classifier employing the histogram of gradients with principal component analysis (HOG/PCA), pocket matching with G-LoSA, molecular docking with Vina, and sequence signature detection with ScanProsite. The performance is assessed with the accuracy (ACC), precision (PPV), sensitivity (TPR), specificity (TNR), and the area under the curve (AUC).

Algorithm	ACC	PPV	TPR	TNR	AUC
DeepDrug3D	0.943	0.951	0.896	0.971	0.986
Pocket volume	0.783	-	-	-	0.512
Pocket shape	0.739	0.654	0.730	0.746	0.824
HOG/PCA	0.689	0.607	0.645	0.719	0.611
ScanProsite	0.722	0.917	0.411	0.970	-
G-LoSA	0.730	0.750	0.589	0.843	0.770
Vina	0.634	0.578	0.648	0.622	0.701

**Table 2:** Performance of various algorithms to classify heme-binding sites. DeepDrug3D is compared to volume- and shape-based approaches, a classifier employing the histogram of gradients with principal component analysis (HOG/PCA), pocket matching with G-LoSA, molecular docking with Vina, and sequence signature detection with ScanProsite. The performance is assessed with the accuracy (ACC), precision (PPV), sensitivity (TPR), specificity (TNR), and the area under the curve (AUC).

Algorithm	ACC	PPV	TPR	TNR	AUC
DeepDrug3D	0.956	0.815	0.909	0.964	0.987
Pocket volume	0.581	-	-	-	0.483
Pocket shape	0.912	0.654	0.730	0.746	0.952
HOG/PCA	0.856	0.616	0.671	0.900	0.611
ScanProsite	0.843	0.990	0.330	0.999	-
G-LoSA	0.891	0.830	0.671	0.958	0.917
Vina	0.602	0.336	0.711	0.569	0.749

Thus far, DeepDrug3D combining deep learning algorithms with interaction-based physicochemical properties of binding sites is shown to outperform approaches employing the volume and shape information alone. Next, we evaluate the benefit of using the voxel representation of pockets in the CNN rather than a more traditional feature vector. Here, features are extracted from voxels with the histogram of gradient (HOG) technique [79], followed by the dimensionality reduction with the principal component analysis (PCA). This combined HOG/PCA approach represents binding sites as 1,024-element feature vectors. The AUC for a CNN trained on the HOG/PCA descriptors is only 0.611 against nucleotide- and heme-binding pockets, thus representing pockets with voxels yields the best performance.

### Comparison to other approaches

**Fig 5:**
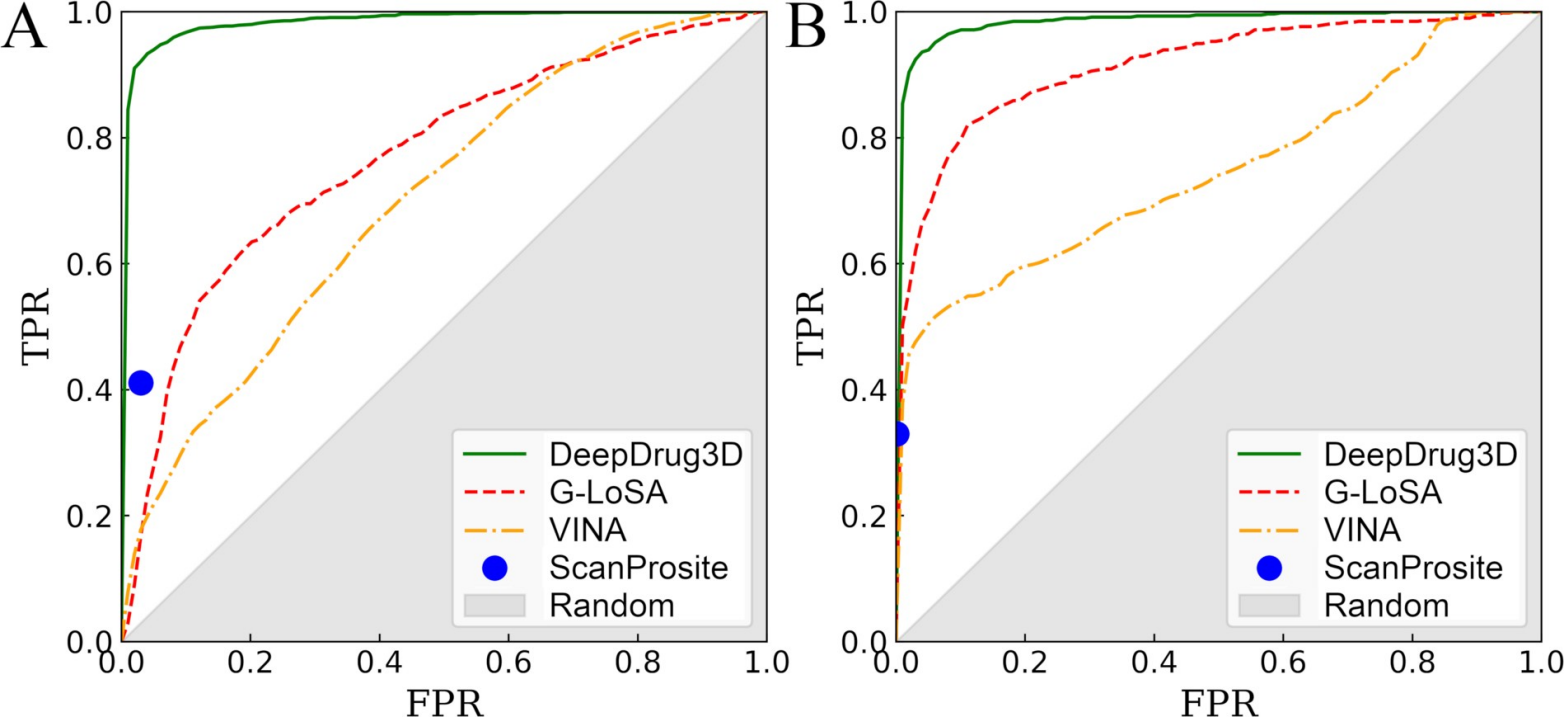
ROC plots evaluating the performance of DeepDrug3D and other methods to classify ligand-binding sites. DeepDrug3D is compared to pocket matching with G-LoSA, molecular docking with Vina, and sequence signature detection with ScanProsite for (**A**) nucleotide- and (**B**) heme-binding pockets. The *x*-axis shows the false positive rate (FPR) and the *y*-axis shows the true positive rate (TPR). The gray area represents a random prediction.

Three different approaches to classify binding pockets are selected for comparative benchmarks on the TOUGH-C1 dataset. The first method, ScanProsite, classifies target proteins based on the presence of PROSITE signatures. Fig 5 and Tables 1 and 2 show that ScanProsite identifies nucleotide- (heme-) binding pockets with a sensitivity of 0.411 (0.330), a precision of 0.917 (0.990), and a specificity of 0.970 (0.999). High precision and specificity values at a notably low sensitivity are typical for short sequence signatures, which are ineffective in detecting those binding sites formed by residues faraway in the sequence [80]. Classifying binding sites based on local structure alignments with G-LoSA yields an accuracy almost as high as that of the shape-based approach. For instance, the AUC for G-LoSA-based classifier is 0.770 for nucleotide- (Fig 5A and Table 1) and 0.917 for heme-binding pockets (Fig 5B and Table 2). Interestingly, inverse virtual screening with Vina can also be used to classify nucleotide- and heme-binding sites. A classifier employing binding affinities predicted by docking yields an AUC of 0.701 for nucleotide- (Fig 5A and Table 1) and 0.749 for heme-binding pockets (Fig 5B and Table 2). Similar to techniques based on local sequence and structure characteristics, ligand docking is more accurate for heme- than nucleotide-binding sites.

These performance differences arise from the fact that heme is more rigid than nucleotides, thus heme-binding sites are structurally more similar to one another than nucleotide-binding sites. Indeed, the average ±standard deviation pairwise root-mean-square deviation (RMSD) calculated according to a procedure described in [28] is 4.4 ±0.7 Å for heme- and 4.8 ±1.0 Å for nucleotide-binding pockets. Furthermore, heme-binding pockets are more dissimilar from control pockets with an RMSD of 6.0 ±1.2 Å compared to nucleotide-binding sites whose RMSD against control pockets is 5.3 ±1.1 Å. Pocket matching with G-LoSA and molecular docking with Vina capitalize on these characteristics resulting a higher classification accuracy for heme-binding sites. Nonetheless, DeepDrug3D employing the voxel representation of ligand-binding sites and deep learning offers a performance that is not only better than other methods, but also more uniform across the TOUGH-C1 dataset.

### Classification performance for additional datasets

**Fig 6:**
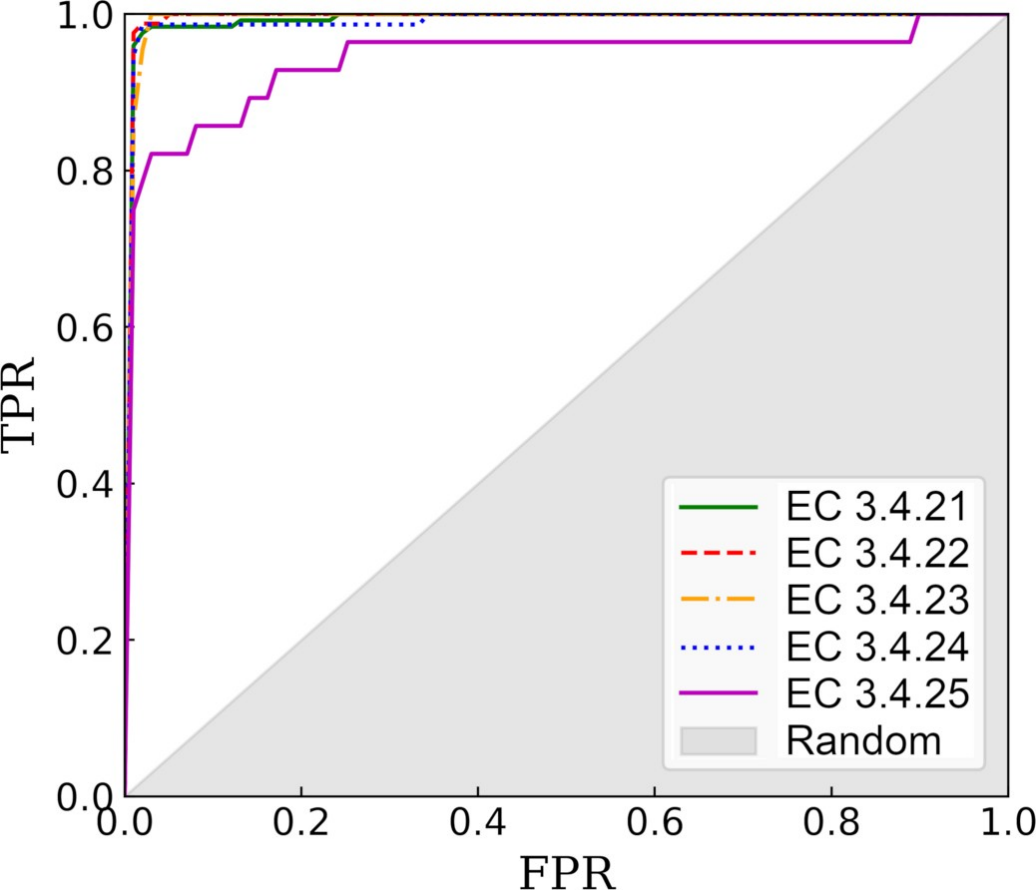
ROC plots evaluating the performance of DeepDrug3D against the peptidase dataset. The performance is assessed individually for five groups of enzymes, serine endopeptidases (EC 3.4.21), cysteine endopeptidases (EC 3.4.22), aspartic endopeptidases (EC 3.4.23), metalloendopeptidases (EC 3.4.24), and threonine endopeptidases (EC 3.4.25). The *x*-axis shows the false positive rate (FPR) and the *y*-axis shows the true positive rate (TPR). The gray area represents a random prediction.

DeepDrug3D trained on nucleotide- and heme-binding pockets against the control dataset is applied to steroid-binding pockets in order to assess the performance of the model on unseen data. Note that the subset of TOUGH-C1 used in training does not include any steroid-like molecules. Encouragingly, 68 out of 69 steroid-binding pockets are classified by DeepDrug3D as “other”, i.e. non-nucleotide- and non-heme-binding. Only a binding pocket for 4-androstene-3-17-dione in glutathione S-transferase A2 (PDB-ID: 2vct, chain A) [81] was incorrectly classified as nucleotide-binding. This misclassification is by narrow margin though, with probabilities of 0.498, 0.032, and 0.469 for nucleotide-, heme-binding, and “other”, respectively. In order to further evaluate the performance of DeepDrug3D, the model is cross-validated against the peptidase and control datasets. In each validation round, one EC group of peptidase enzymes is excluded from training and the model accuracy is assessed for these omitted targets. Fig 6 shows ROC plots calculated for individual peptidase groups. DeepDrug3D accurately classifies peptidase enzymes with AUC values of 0.996, 0.998, 0.996, 0.994, and 0.943 for serine endopeptidases (EC 3.4.21), cysteine endopeptidases (EC 3.4.22), aspartic endopeptidases (EC 3.4.23), metalloendopeptidases (EC 3.4.24), and threonine endopeptidases (EC 3.4.25), respectively. The results obtained for steroid-binding proteins and peptidase enzymes demonstrate that DeepDrug3D generalizes well from the training data to unseen data, which is critical to avoid overprediction in real applications.

### CAM analysis of binding pockets

**Fig 7:**
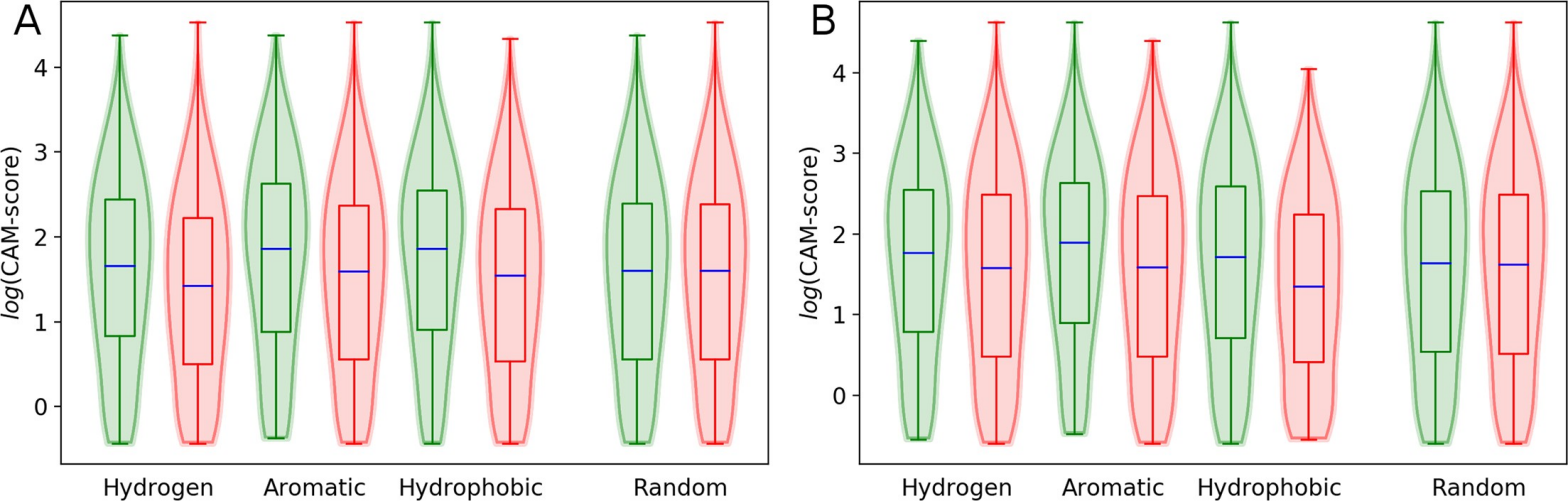
Distribution of class-activation map (CAM) scores for specific ligand-protein interactions. Three interaction types, hydrogen bonds, aromatic and hydrophobic contacts are reported by the LPC program for (**A**) nucleotide- and (**B**) heme-binding pockets. For each interaction type, grid points are divided into two groups, those points in close proximity to residues forming a particular contact and the remaining points that are closer to residues not forming these interactions. The last pair of violins are plotted for grid points randomly assigned into two groups, irrespectively of any ligand-protein interactions. Horizontal blue lines represent median values, and whiskers extend to the most extreme non-outlier data points.

The CAM analysis reveals distinct regions of binding sites that are important for their accurate classification with the CNN. As illustrated in Fig 3, we map these highly discriminative locations to binding residues by calculating CAM-scores from the top 1% of CAM values. Interestingly, as many as 74% of residues assigned CAM-scores in nucleotide-binding and 57% in heme-binding pockets form interactions with ligands according to the LPC software [65]. Since the CAM analysis is conducted for protein structures alone, without any information on bound ligands, DeepDrug3D quite accurately detects binding residues. Next, we examine whether those amino acids forming specific contacts with ligands are assigned higher CAM-scores than other residues. The distribution of CAM-score values for three types of interactions, hydrogen bonds, aromatic and hydrophobic contacts, are shown in Fig 7. For example, the first two violins in Fig 7A are calculated for residues forming hydrogen bonds (green) and not forming hydrogen bonds with nucleotides (red). The equivalent distribution of CAM-score values for heme-binding residues are presented in Fig 7B. The Mann-Whitney U test indicates significant differences between the two groups of residues for each interaction type with *p*-values for hydrogen bonds, aromatic and hydrophobic contacts of 3.19 × 10^−37^, 1.03 × 10^−10^, and 8.39 × 10^−45^ (nucleotide-binding pockets) and 2.73 × 10^−05^, 5.15 × 10^−9^, and 6.51 × 10^−26^ (heme-binding pockets), respectively. For comparison, *p*-values for binding residues randomly divided into two groups irrespectively of any interaction type are 0.22 for nucleotide- and 0.18 for heme-binding pockets (the last two violins in Fig 7A and 7B).

The CAM analysis indicates that DeepDrug3D learns from patterns of specific molecular interactions formed by various ligands and proteins across the TOUGH-C1 dataset. In this section, we look into those interactions identified by the CAM as important for the accurate classification with the CNN. Interestingly, Table 3 shows that 70.7% of CAM designated residues in nucleotide-binding pockets form hydrogen bonds with bound ligands. These results agree with a number of studies on nucleotide-binding proteins. For instance, a comprehensive analysis of 233 X-ray complex structures of protein kinases reveals that hydrogen bonding is the key interaction for nucleotide and inhibitor binding in the kinase family with both strong and weak hydrogen bonds equally important [82]. Further, the formation of hydrogen bonds was suggested to be essential for nucleotide binding on account of a buried amino group of the adenine ring in the complexed state [83]. Not surprisingly, these distinct interaction patterns are commonly exploited in drug discovery. Multiple hydrogen bonds within the hinge region of protein kinases serving as the anchor to bind ATP are generally indispensable for binding of potent enzyme inhibitors [84]. The potency of an ATP-competitive inhibitor of CDK2 was significantly increased by forming additional hydrogen bonds between the compound and binding residues in the enzyme [85]. These observations explain the high content of residues forming hydrogen bonds within strongly discriminative regions of nucleotide-binding pockets identified by the CAM.

**Table 3:** Percentage of binding residues forming specific interactions with ligands. Binding residues are identified by the class-activation map analysis to be part of the highly discriminative regions of nucleotide- and heme-binding pockets. Three types of interactions reported by the LPC program are considered, hydrogen bonds, aromatic and hydrophobic contacts.

Pocket type	Hydrogen	Aromatic	Hydrophobic
Nucleotide-binding	70.7%	4.8%	21.3%
Heme-binding	23.8%	12.9%	75.3%

In contrast, Table 3 reports that as many as 75.3% of residues designated by the CAM as important for heme binding are involved in hydrophobic interactions and 12.9% form aromatic interactions. Indeed, a study examining the effects of pocket hydrophobicity in myoglobin revealed that non-polar interactions contribute to a control mechanism for the binding of heme [86]. Moreover, the analysis of hydrophobic interactions in porphyrin-containing proteins showed that heme-protein complexes are stabilized by the non-polar side-chains of binding residues [87]. A survey of a non-redundant set of 125 heme-binding proteins belonging to 31 structural folds concluded that mainly non-polar and aromatic residues create a hydrophobic environment suitable for the heme ring structure [88]. Because of the important role of hydrophobic interactions in heme binding, the analysis of physicochemical properties of protein residues was demonstrated to effectively identify heme-binding proteins [89]. In agreement with these studies, the CAM analysis reveals that the discriminative regions of heme-binding pockets are predominantly composed of non-polar amino acids.

### Case studies

**Fig 8:**
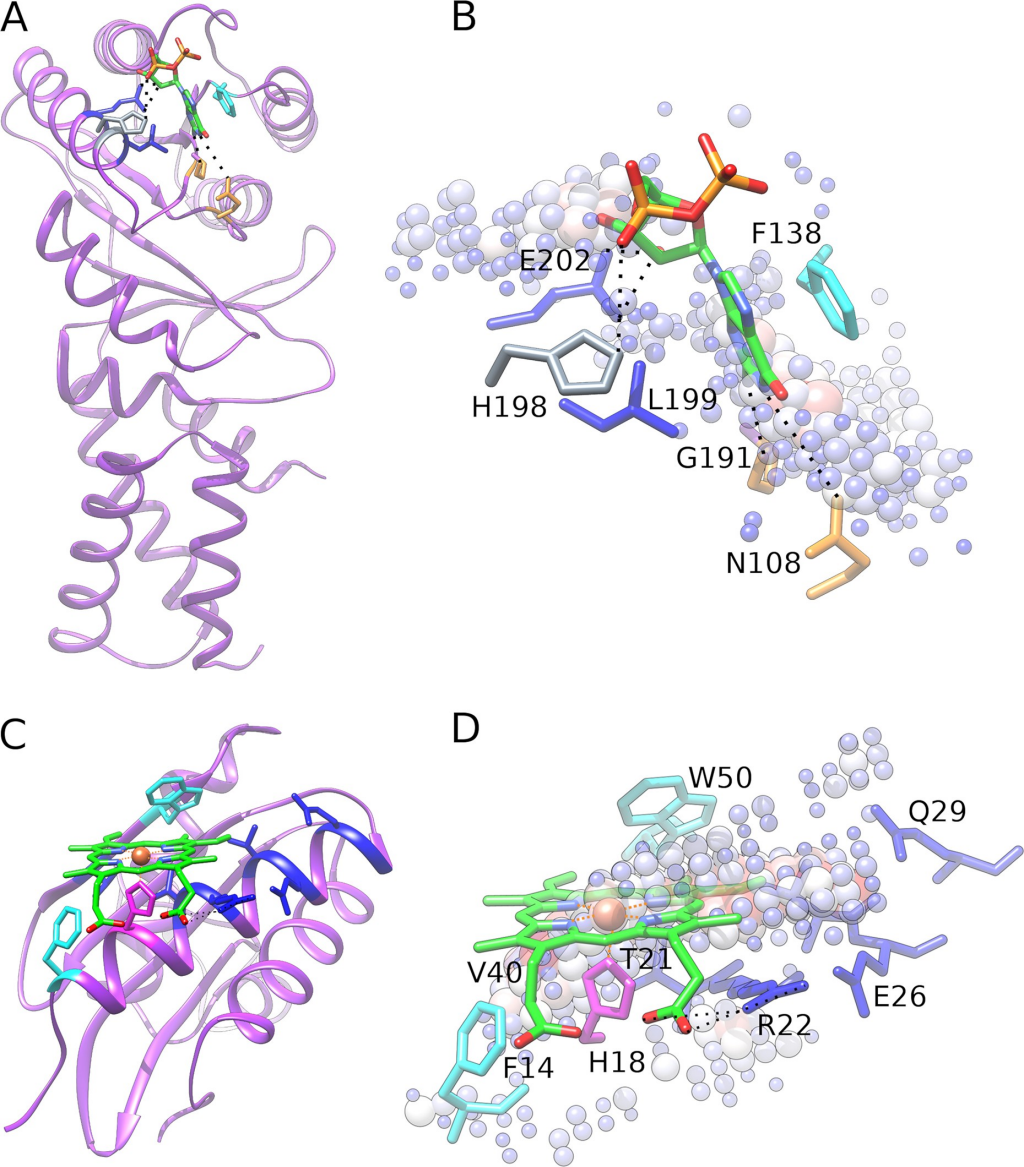
Two examples of accurately classified ligand-binding pockets. (**A** and **B**) A GDP-binding protein, the signal recognition particle receptor ftsY from *E*. *coli*, and (**C** and **D**) a heme-binding protein, the C-terminal domain of the *S*. *enterica* PduO protein. (**A** and **C**) Experimental complex structures and (**B** and **D**) close-ups of binding sites with high-scoring class-activation map (CAM) grid points. GDP and heme are shown as green sticks colored by atom type (C–green, O–red, N–blue, P and Fe–orange), whereas grid points are represented by spheres whose size and color depend on CAM values according to the scale shown in Fig 3. Hydrogen bonds are indicated by dashed black lines. Selected binding residues are labeled and colored by the interaction type (hydrogen bond–orange, aromatic–gray, hydrophobic–blue, aromatic and hydrophobic–cyan, hydrogen bond, aromatic and hydrophobic–magenta).

Two representative examples are presented in Fig 8 to further elucidate how the CAM analysis can be used to identify discriminative regions of binding sites. The first protein is the GTPase domain of the signal recognition particle receptor FtsY from *Escherichia coli* (PDB-ID: 4c7o, chain D) [90]. Fig 8A shows the crystal structure of FtsY bound to GDP, in which the ligand forms hydrogen bonds with N108, G191, H198 and E202, hydrophobic interactions with F138, L199 and E202, and aromatic interactions with F138 and H198. It is evident from Fig 8B that binding residues involved in specific interactions according to LPC are located within highly discriminative regions. For instance, H198 forming an aromatic interaction with GDP has a CAM-score of 26.4 and it is close to several blue spheres. Further, hydrogen-bonded residues N108 and E202, assigned high CAM-scores of 28.9 and 39.1, respectively, are in a short distance from large red spheres. Based on these interactions, DeepDrug3D is able to correctly classify FtsY as nucleotide-binding with a probability of 0.685, whereas the probability of heme-binding is 0.280 and the “other” class is 0.035. In general, the observation that residues forming hydrogen bonds with ligands are located within strongly discriminative regions is in line with previous studies, in which a limited number of hydrogen bond patterns in nucleotide-bound complexes is utilized as an effective classification scheme [91].

The second example is the C-terminal domain of a protein PduO (PduOC) playing an important role in the catabolism of 1,2-propanediol in the pathogenic bacterium *Salmonella enterica* (PDB-ID: 5cx7, chain E) [24]. Fig 8C presents the structure of PduOC co-crystallized with heme, whose binding mode significantly differs from those in other members of this protein family. Heme interacts with PduOC by forming hydrogen bonds with H18 and R22, aromatic interactions with F14, H18, W50 and number of hydrophobic contacts with F14, H18, W21, R22, V25, E26, V48 and W50. Many of these residues are assigned high CAM-scores, for instance, essential for heme binding H18 has a CAM-score of 64.9 and it is located near large red spheres (Fig 8D). R22 is another important residue interacting with the heme propionate group via a double salt bridge. This amino acid, assigned a CAM-score of 78.1, is part of a highly discriminative region of the binding site as well. PduOC is particularly interesting because despite the novel binding mode of heme, the cross-validated CNN unambiguously classifies PduOC as heme-binding with a probability of 0.960, whereas the probability of nucleotide-binding is 0.027 and the “other” class is 0.013. FtsY and PduOC discussed here as case studies not only exemplify accurate classifications with DeepDrug3D, but also indicate that the identified important regions are potentially valuable in rational drug design.

## Availability and future directions

DeepDrug3D is designed to effectively categorize ligand-binding pockets in protein structures with primary applications in the identification of physiological ligands for orphan receptors, the exploration of distant evolutionary relationships between proteins, and modern drug development focusing on polypharmacology, drug repositioning and the analysis of side-effects. It is freely available to the academic community as an open-source software package at https://github.com/pulimeng/DeepDrug3D. This repository also includes a complete documentation with installation and execution instructions, and sample input and output files. In addition to the code, the TOUGH-C1 dataset is available at https://osf.io/enz69/.

In the near future, the repertoire of pocket types handled by DeepDrug3D will be expanded. This expansion will inevitably create a rare class recognition problem in deep learning because various ligands and pockets are non-uniformly covered by experimental structures in the PDB. On that account, we plan to explore several strategies commonly used in machine learning to overcome difficulties caused by the class imbalance in the training data. A popular tactics is to even-up the classes by over- and under-sampling in order to build a more balanced dataset to train the predictive model. Further, the training data will be augmented with “synthetic samples”, i.e. highly confident computer-generated models of drug-protein complexes. Here, we will use *e*ModelBDB [8], a recently developed database of 200,005 comparative models of drug-bound proteins based on interaction data obtained from the Binding Database [92]. Other developments will include conducting rigorous benchmarks against the homology models of target proteins with computationally annotated ligand-binding sites. Considering the successful application of other methods developed by our group, *e*FindSite [93] and *e*MatchSite [34], in large-scale projects utilizing protein models [37, 38], as well as a remarkable robustness of deep learning [94, 95], we expect DeepDrug3D to maintain its high classification accuracy even against noisy data. A high tolerance to structural distortions in computer-generated protein models will open up the possibility to deploy DeepDrug3D in large, across-proteome projects.
